# Supplementary material for: The Immunological Factors Predisposing to Severe Covid-19 Are Already Present in Healthy Elderly and Men
Source: Front Immunol. 2021 Aug 9;12:720090. doi: 10.3389/fimmu.2021.720090 (PMC8380832; doi:10.3389/fimmu.2021.720090)
Supplement: Supplementary file 1 [file DataSheet_1.docx]

**Supplementary Figures**


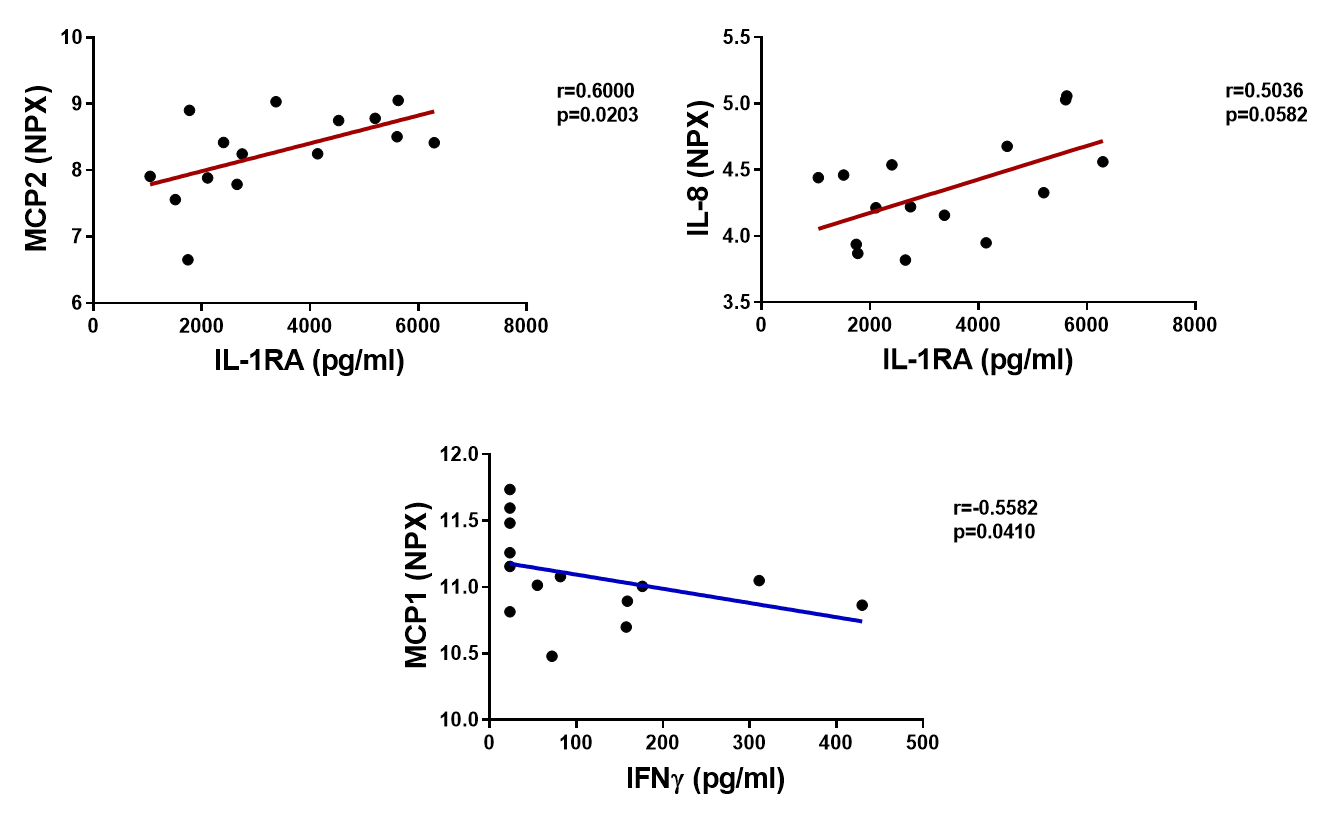


**Supp. Figure 1.** Correlation between baseline plasma protein levels and cytokine productions against *in vitro* SARS-CoV-2 stimulation in healthy individuals. The x-axis shows the cytokine productions of PBMCs after stimulation with heat-inactivated SARS-CoV-2 while the y-axis demonstrates the baseline plasma protein levels of healthy individuals. Red indicates a positive correlation whereas blue indicates a negative correlation. NPX: normalized protein expression, r = Spearman correlation coefficient, n=14-15.


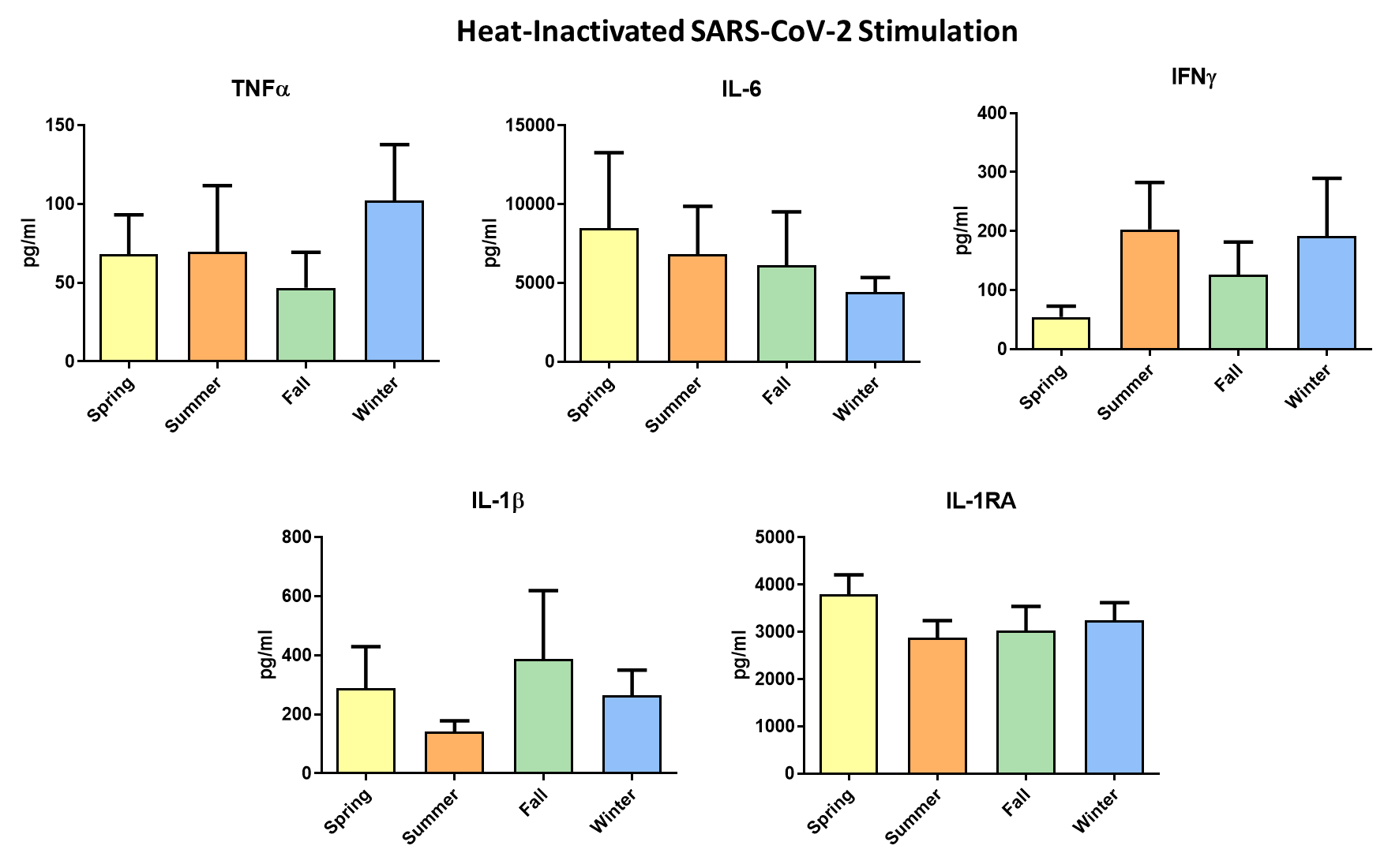
**Supp. Figure 2.** The cytokine productions of PBMCs that were stimulated with heat-inactivated SARS-CoV-2. The PBMCs from healthy individuals were collected and frozen at different times of the year. n=7-20. Error bars depict the standard error of the mean (SEM).

**
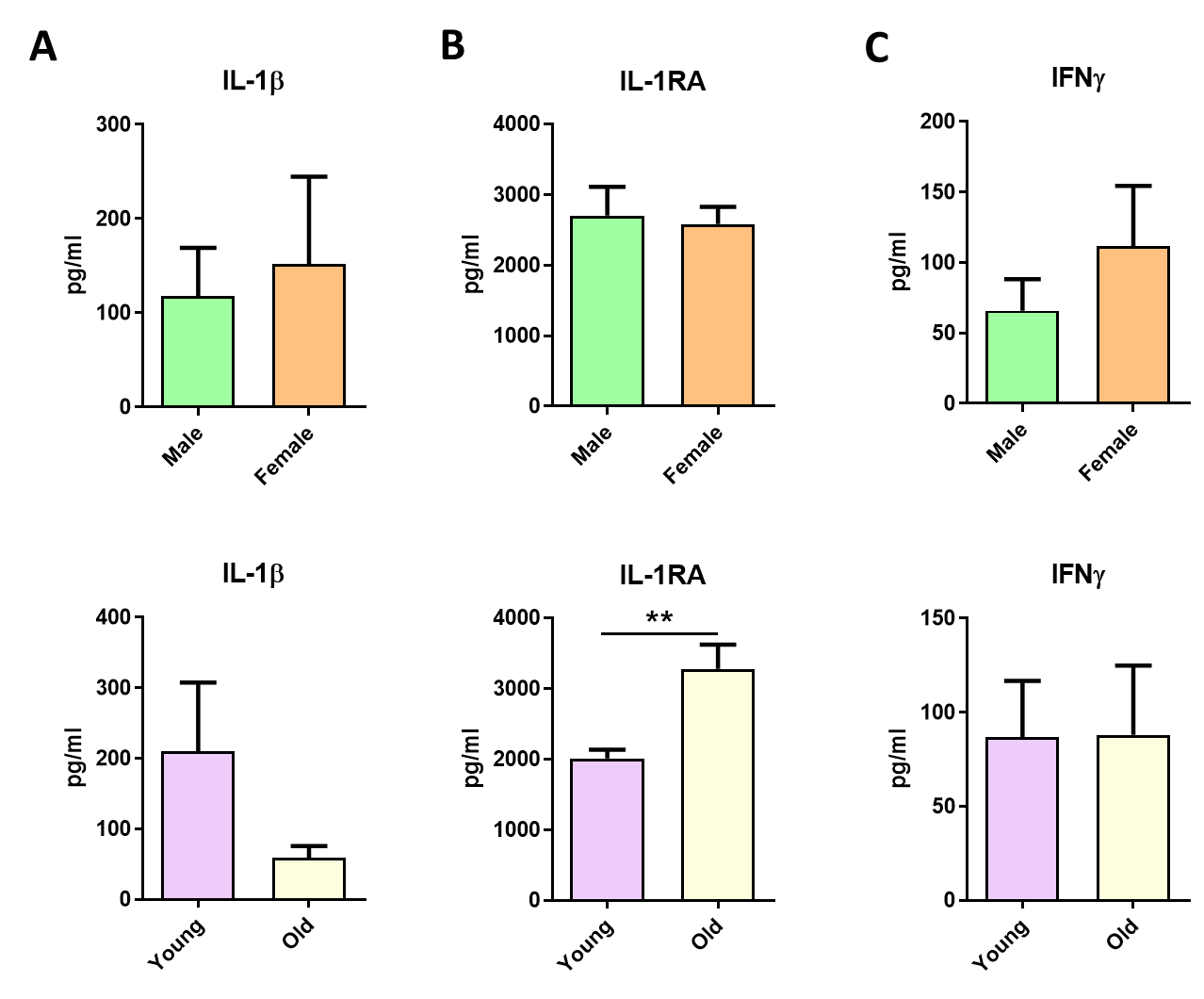
**

**Supp. Figure 3.** Basal cytokine responses of healthy individuals in the absence of any stimulus. Responses were compared between males and females (upper row) and between young and old individuals (lower row). The graphs show the yearly average production. (A) IL-1β and (B) IL-1RA cytokine levels were measured after 24 hours, while (C) IFNγ was measured after 5 days. **p ≤ 0.01. n=8-10. Error bars depict the SEM.

**
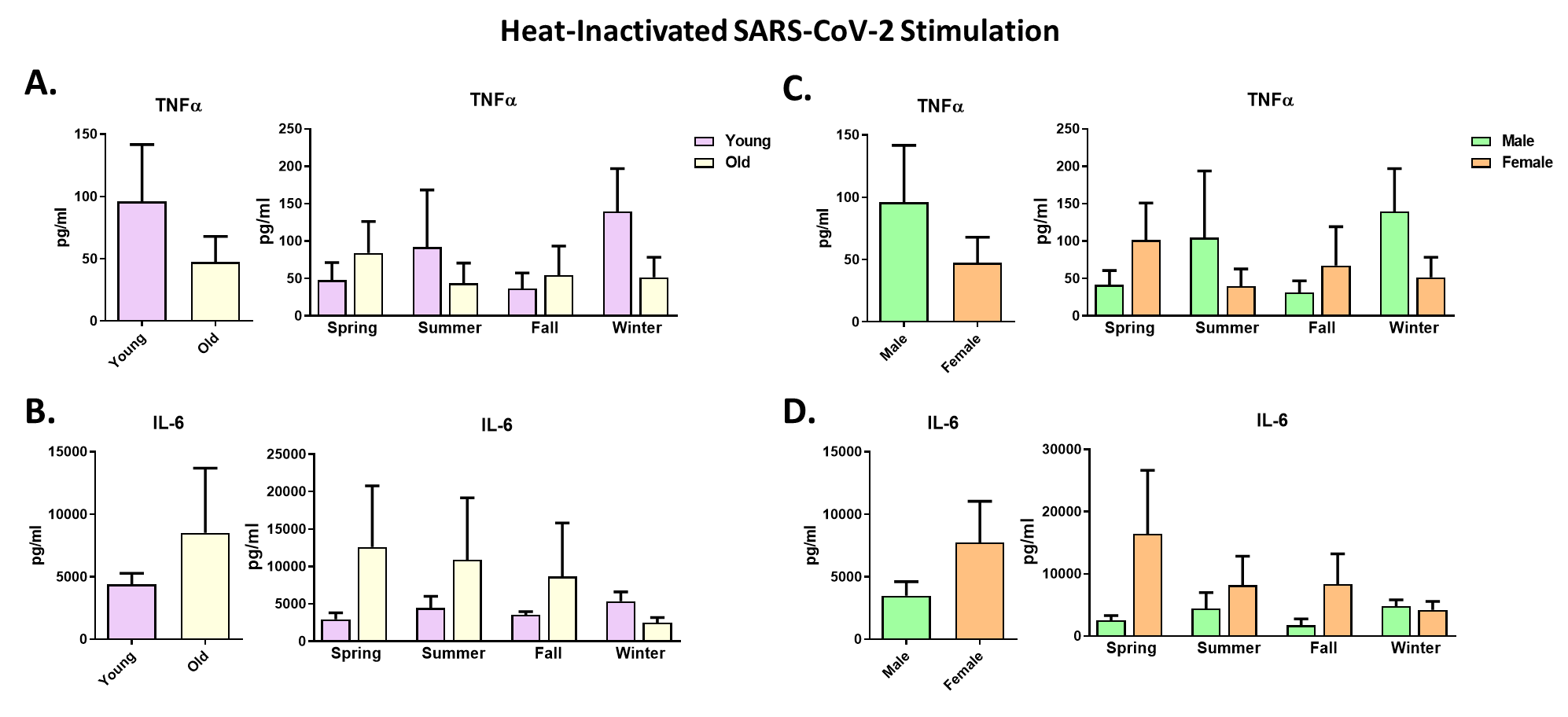
**

**Supp. Figure 4.** Cytokine responses against heat-inactivated SARS-CoV-2 in healthy individuals. Each panel's left graphs show the yearly average production, while the right graphs demonstrate cytokine production in every season. Responses were compared between young and old individuals (A-B) and between males and females (C-D). Cytokine levels were measured after 24 hours. n=3-8. Error bars depict the SEM.

**
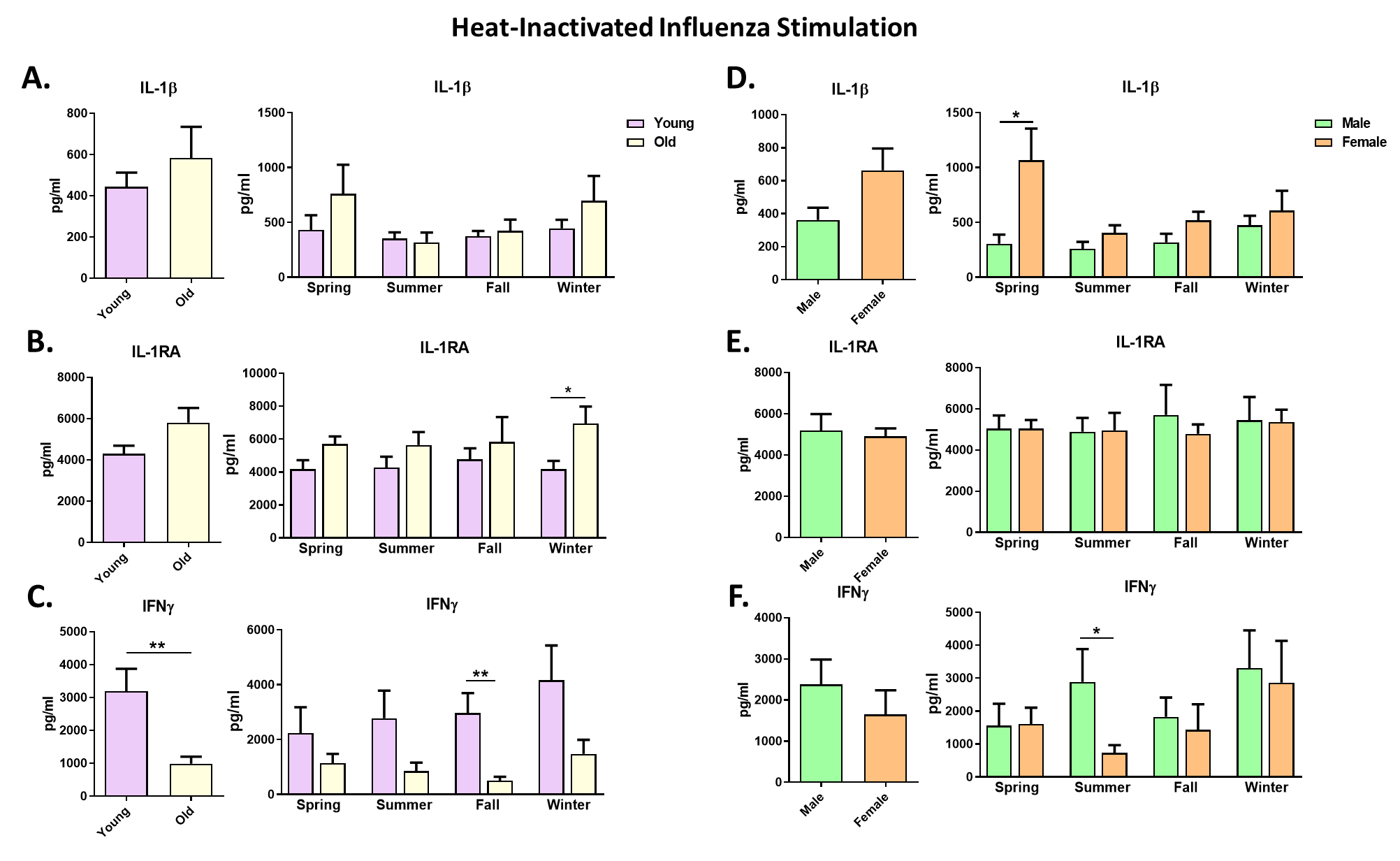
**

**Supp. Figure 5.** Immune responses against heat-inactivated influenza A (H1N1) in healthy individuals. Each panel's left graphs show the yearly average production, while the right graphs demonstrate cytokine production in every season. Responses were compared between young and old individuals (A-C) and between males and females (D-F). IL-1β and IL-1RA cytokine levels were measured after 24 hours, while IFNγ was measured after 5 days. *p ≤ 0.05, **p ≤ 0.01. n=5-10. Error bars depict the SEM.

**Supplementary Tables**

**Supplementary Table 1:** Plasma proteins and immune cell populations linked with severe COVID-19.

| **Reference** | **Severe COVID-19 Immune Profile** |
| --- | --- |
| **Janssen et al. (2021)**  ***J Infect Dis*** | Higher CRP, D-dimer, TNFα, IL-6, IL-8, IL-18, HGF, CCL3, CCL19, CCL20, ENRAGE, MCP3, VEGFA, and CD40.  Lower SCF, DNER, TRAIL, TRANCE, TNFB, VEGFD, and HLA-DR expression. |
| **Qin et al. (2020)**  ***Clin Infect Dis*** | Higher TNFα, IL-6, IL-8, IL-10, CRP, ferritin, memory CD4+ T cells, and neutrophils.  Lower lymphocytes, T cells, CD4+ T cells, and Tregs. |
| **Huang et al. (2020) *Cytometry A*** | Lower lymphocytes, CD4+ T cells, CD8+ T cells, CD56+ NK cells and B cells. |
| **Yang et al. (2020)**  ***J Allergy Clin Immunol*** | Higher IP-10, MCP-3, HGF, MIP-1α, IL-1RA, and CSF1. |
| **Bergamaschi et al. (2021)**  ***MedRxiv*** | Higher plasmablasts, classical monocytes, and neutrophils.  Lower CD4+ naïve, CD4+ central memory, CD4+ effector memory, Tregs, CD4+ follicular helper, CD8+ naïve, CD8+ effector memory, B cells, transitional B cells, memory B cells, pDCs, and non-classical monocytes. |
| **Chen et al. (2020)**  ***J Clin Invest*** | Higher lactate dehydrogenase, D-dimers, CRP, IL-6, IL-10, TNFα, and neutrophils.  Lower lymphocytes, CD4+ T cells, CD8+ T cells, and naïve T regs. |
| **Huang et al. (2020)**  ***Lancet*** | Higher IL-2, IL-7, IL-10, IP-10, MCP-1, MIP1α, TNFα, and G-CSF. |

**Supplementary Table 2.** List of immune cell types and circulating proteins investigated in the study. These have been previously linked to COVID-19 severity.

| IMMUNE CELL TYPES |  | CIRCULATING PROTEINS |
| --- | --- | --- |
| Leukocytes |  | Interferon gamma (IFNγ) |
| Lymphocytes |  | Interleukin 6 (IL-6) |
| Neutrophils |  | Interleukin 7 (IL-7) |
| Monocytes |  | Interleukin 8 (IL-8) |
| Classical Monocytes |  | Interleukin 10 (IL-10) |
| Intermediate Monocytes |  | Interleukin 18 (IL-18) |
| Non-classical Monocytes |  | Monocyte chemoattractant protein 1 (MCP-1) |
| Natural Killer (NK) Cells |  | Monocyte chemoattractant protein 2 (MCP-2) |
| CD56^dim^ NK Cells |  | Chemokine (C-C motif) ligand 3 (CCL3) |
| CD56^bright^ NK Cells |  | Chemokine (C-C motif) ligand 4 (CCL4) |
| Natural Killer T Cells |  | Chemokine (C-C motif) ligand 19 (CCL19) |
| T Cells |  | Chemokine (C-C motif) ligand 20 (CCL20) |
| Naïve T Cells |  | Chemokine (C-X-C motif) ligand 9 (CXCL9) |
| Memory T Cells |  | Chemokine (C-X-C motif) ligand 10 (CXCL10) |
| CD4^+^ T Cells |  | Tumor necrosis factor (TNF) |
| Naïve CD4^+^ T Cells |  | Tumor necrosis factor beta (TNFB) |
| Effector CD4^+^ T Cells |  | TNF-related activation-induced cytokine (TRANCE) |
| Effector Memory CD4^+^ T Cells |  | TNF-related apoptosis-inducing ligand (TRAIL) |
| Central Memory CD4^+^ T Cells |  | TNF-related weak inducer of apoptosis (TWEAK) |
| CD8^+^ T Cells |  | Latency associated peptide - transforming growth factor beta (LAP-TGFB1) |
| Naïve CD8^+^ T Cells |  | Osteoprotegerin (OPG) |
| Effector CD8^+^ T Cells |  | Colony stimulating factor 1 (CSF1) |
| Effector Memory CD8^+^ T Cells |  | Stem cell factor (SCF) |
| Central Memory CD8^+^ T Cells |  | Hepatocyte growth factor (HGF) |
| Regulatory T cells (Tregs) |  | Vascular endothelial growth factor alpha (VEGFA) |
| Naïve Tregs |  | Extracellular newly identified RAGE-binding protein (EN-RAGE) |
| Memory Tregs |  | Delta and Notch-like epidermal growth factor-related receptor (DNER) |
| B Cells |  | Programmed death-ligand 1 (PD-L1) |
| Plasmablasts |  |  |
| Naïve B Cells |  |  |
| Mature Naïve B Cells |  |  |
| Transitional B Cells |  |  |
| Natural Effector B Cells |  |  |
| Memory B Cells |  |  |
| Class-switched Memory B Cells |  |  |
| Non-class-switched Memory B Cells |  |  |

**Supplementary Table 3.** Demographics of the 20 individuals selected from Cohort 1 for seasonality analysis. Age and BMI are depicted as mean ± standard deviation.

| Group (n=5) | Age | Body mass index (BMI) |
| --- | --- | --- |
| Young male | 23.2 ± 3.11 | 24.46 ± 1.47 |
| Old male | 64.6 ± 3.65 | 24.39 ± 2.46 |
| Young female | 23.8 ± 3.11 | 22.40 ± 1.63 |
| Old female | 60.8 ± 6.22 | 24.07 ± 3.70 |
